# A Novel Biomarker Panel Examining Response to Gemcitabine with or without Erlotinib for Pancreatic Cancer Therapy in NCIC Clinical Trials Group PA.3

**DOI:** 10.1371/journal.pone.0147995

**Published:** 2016-01-25

**Authors:** David B. Shultz, Jonathan Pai, Wayland Chiu, Kendall Ng, Madeline G. Hellendag, Gregory Heestand, Daniel T. Chang, Dongsheng Tu, Malcolm J. Moore, Wendy R. Parulekar, Albert C. Koong

**Affiliations:** 1 Princess Margaret Cancer Centre, Toronto, Ontario, Canada; 2 School of Medicine, University of California San Francisco, San Francisco, United States of America; 3 Department of Radiation Oncology, Stanford University School of Medicine, Stanford, CA, United States of America; 4 Kaiser Permanente Medical Center, San Francisco, United States of America; 5 Moores Cancer Center, University of California San Diego, La Jolla, CA, United States of America; 6 NCIC Clinical Trials Group, Queen's University, Kingston, Canada; 7 British Columbia Cancer Agency, Vancouver, British Columbia, CA, United States of America; Queen Mary Hospital, HONG KONG

## Abstract

**Purpose:**

NCIC Clinical Trials Group PA.3 was a randomized control trial that demonstrated improved overall survival (OS) in patients receiving erlotinib in addition to gemcitabine for locally advanced or metastatic pancreatic cancer. Prior to therapy, patients had plasma samples drawn for future study. We sought to identify biomarkers within these samples.

**Experimental Design:**

Using the proximity ligation assay (PLA), a probe panel was built from commercially available antibodies for 35 key proteins selected from a global genetic analysis of pancreatic cancers, and used to quantify protein levels in 20 uL of patient plasma. To determine if any of these proteins levels independently associated with OS, univariate and mulitbaraible Cox models were used. In addition, we examined the associations between biomarker expression and disease stage at diagnosis using Fisher's exact test. The correlation between Erlotinib sensitivity and each biomarkers was assessed using a test of interaction between treatment and biomarker.

**Results and Conclusion:**

Of the 569 eligible patients, 480 had samples available for study. Samples were randomly allocated into training (251) and validation sets (229). Among all patients, elevated levels of interleukin-8 (IL-8), carcinoembryonic antigen (CEA), hypoxia-inducible factor 1-alpha (HIF-1 alpha), and interleukin-6 were independently associated with lower OS, while IL-8, CEA, platelet-derived growth factor receptor alpha and mucin-1 were associated with metastatic disease. Patients with elevated levels of receptor tyrosine-protein kinase erbB-2 (HER2) expression had improved OS when treated with erlotinib compared to placebo. In conclusion, PLA is a powerful tool for identifying biomarkers from archived, small volume serum samples. These data may be useful to stratify patient outcomes regardless of therapeutic intervention.

**Trial Registration:**

ClinicalTrials.gov NCT00040183

## Introduction

Pancreatic ductal adenocarcinoma (PDCA) is an aggressive malignancy with a poor prognosis. Patients diagnosed with resectable tumors are potentially curable, however locally advanced (LA) or metastatic PDCA is uniformly fatal.[1, 2]Improved biomarkers are needed to refine the therapeutic management of advanced PDCA and improve outcomes.

Validated prognostic biomarkers for pancreatic ductal adenocarcinoma (PDCA) are limited. CA 19–9, the most established biomarker for PDCA, has a sensitivity and specificity for pancreatic cancer of approximately 80% and 90%, respectively [3] and in some scenarios levels are predictive of chemotherapy or radiation responses [4, 5]. However, CA 19–9 can be falsely elevated in patients with obstructive liver disease or pancreatitis, and falsely negative in patients who lack Lewis-antigen glycosyltransferase (5–10% of the population). In addition, CA 19–9 levels provide limited insight into the biologic functions of PDCA that might direct systemic therapy. Therefore, better biomarkers are needed to guide patient care.

PA.3 was a randomized phase III trial conducted by the NCIC Clinical Trials Group that demonstrated improved survival in patients treated with erlotinib plus gemcitabine compared to gemcitabine alone, in which, EGFR expression was not predictive of a response to erlotinib [6]. In this current study, in pre-treatment plasma samples from patients enrolled on PA.3, we sought to identify biomarkers that were prognostic of survival as well as predictive for a response to erlotinib.

## Methods

Plasma samples were obtained from 480 of 569 patients enrolled on NCIC clinical trials group (CTG) PA.3 (Clinical.Trials.gov Identifier NCT00040183), a double blinded international, phase III trial of erlotinib (235) versus placebo plus gemcitabine (245) patients with locally advanced or metastatic pancreatic adenocarcinoma [6]. Samples were obtained at the time of enrollment. The primary end point for this trial was OS. The study described in this manuscript was approved by the Stanford Research Compliance Office: IRB #5136 (protocol 27492). At the time of enrollment on PA.3, patient’s informed written consent was obtained and, under approval from our own research compliance office, we did not obtain seperate informed written or verbal consent from patients for our study.

Prior to laboratory analysis, samples were randomly assigned to a training (251 total, 129 received erlotinib) or a validation (229 total, 106 received erlotinib) cohort. The proximity ligation assay (PLA) [7] was used to measure the relative concentration of 35 biomarker proteins (Table 1). In brief, PLA probe sets were added to blocked serum and incubated at 37°C for two hours. Next, ssDNA splints with ligase were added robotically (Velocity11, Agilent Technologies) and incubated at 30°C for 15 minutes. Finally, Uracil-DNA Excision Mix (Epicentre) was added, and the resulting DNA was amplified using PCR with Platinum Taq (Invitrogen) and the PCR product was then amplified with iTaq with SYBR (Bio-Rad) using quantitative PCR (Model 7500, Applied Biosystems) to determine the relative concentrations of each potential biomarker.

**Table 1:** 

CXCL6	Chemokine (C-X-C motif) ligand 6
CEA	Carcinoembryonic antigen
CA 19–9	Carbohydrate assocaited antigen 19–9
HIF1A	Hypoxia inducible factor 1
SPARC	Secreted protein, acidic, cysteine rich
MMP1	Matric metallopeptidase 1
EGFR	Epidermal growth factor receptor
IL6	Interleukin 6
IGFBP3	Insulin like growth facotor binding protein 3
OPN	Secreted phosphoprotein 1
PDK1	Pyruvate Dehydrogenase 1
GAS6	Growth arrest specific 1
MMP7	Matric metallopeptidase 7
IL8	Interleukin 8
IGF2	Insulin-like growth factor 2
CXCL9	Chemokine (C-X-C motif) ligand 9
MSLN	Mesothelin
Reg4	Regenerating islet-derived family, member 4
IL7	Interleukin 7
PDGFRA	Platelet-derived growth factor receptor, alpha
IGFBP2	Insulin like growth facotor binding protein
CPA-1	Carboxypeptidase A1
VegefD	Vascular endothelial growth factor D
IL12	Interleukin 12
CXCL-10	Chemokine (C-X-C motif) ligand 10
HER2	erb-b2 receptor tyrosine kinase 2
BMP-2	Bone morphogenetic protein 2
Trappin 2	Peptidase inhibitor 3, skin-derived
MMP-2	Matric metallopeptidase 1
CXCL-1	Chemokine (C-X-C motif) ligand 1
Axl	AXL receptor tyrosine kinase
PF4	Platelet factor 4
TGFB1	Transforming growth factor, beta 1
MUC-1	Mucin 1, cell surface associated
MMP-3	Matric metallopeptidase 3

Biomarker probes were developed for prior studies and additional ones were synthesized for this study.[7, 8] Potential biomarkers were identified through a global genetic analysis [9] and consisted of a comprehensive literature search to identify published datasets, followed by an assessment of whether potential biomarkers were expressed on either the cell surface or in the plasma, and finally whether biomarkers were specifically elevated in pancreatic cancer versus chronic pancreatitis. Of the potential candidates identified in that publication, 320 were confirmed to be excreted and present at elevated levels in the serum of patients. Next, we obtained a gene expression analysis dataset in pancreatic ductal adenocarcinoma from the Gene Expression Omnibus [10, 11] and after identifying the levels of gene expression for the 320 putative biomarkers of pancreatic cancer described above, we used a k-means clustering to identify 13 separate sub-categories. From each of these, we selected 5 biomarkers to be included in our panel, for a total of 65 probes. Several additional biomarkers were identified by direct literature search. Because commercially available antibodies for many of the biomarkers identified were unavailable, less than half of the potential PLA probes were built (Table 1).

Biomarker levels were dichotomized into high and low categories based on whether they were greater than the median. We next tested for a statistically significant association with overall survival (OS) in both the training and validation cohorts using Cox regression models. Age (<70 vs. ≥70), sex (female vs. male), race (white vs. non-white), ECOG performance status (0–1 vs. 2), pain intensity (≤20 vs. >20), and disease stage (III vs. IV) were other covariates included in the multivariable regression models. Each biomarker was analyzed individually using the Cox models including the same set of covariates listed above. In a separate analysis, we examined whether biomarker expression (above or not above the median) was associated with disease stage. Biomarkers whose expression was significantly associated with stage were determined using Fisher’s exact test. Finally, for each biomarker, we tested its effect in predicting for improved survival due to erlotinib using a multivariable Cox model including an additional interaction term between the treatment (erlotinib vs. placebo) and the binary biomarker expression level.

## Results

**Fig 1:**
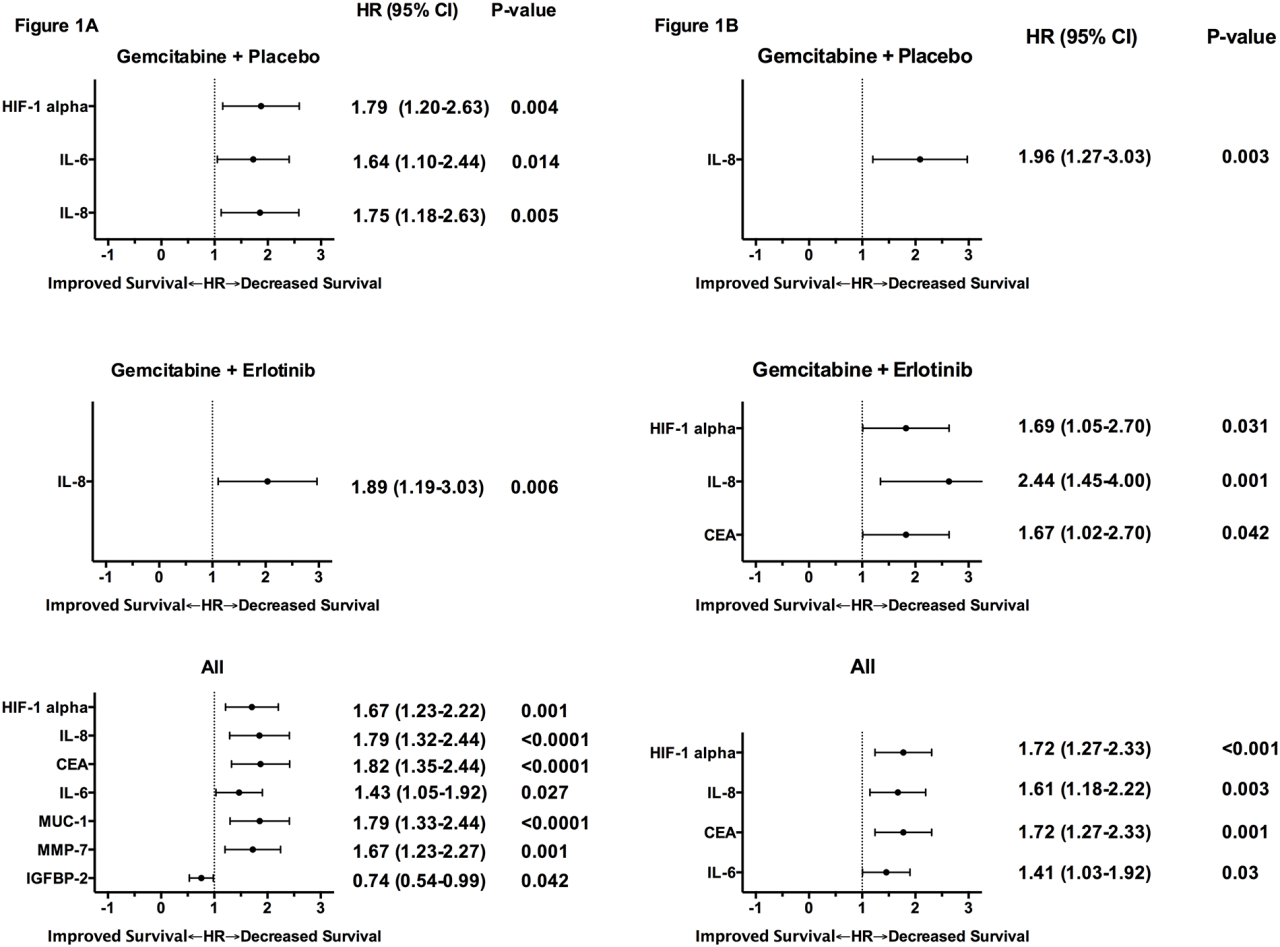
Univariate (A) and multivariable (B) analyses of the association between biomarker concentration, according to median levels, and overall survival. Hazard ratios (HR) (of high to low biomarker concentration) and p-values are shown for biomarkers that were significantly associated (p<0.05) with survival. Results shown are from the validation cohorts.

Proteins whose concentrations, after dichotomizing based on being greater than median, associated significantly (p<0.05) with OS in both the training and validation cohort on univariate or multivariable analysis are presented in Fig 1A and 1B, respectively. Results shown are for patients in the validation cohort. As shown, on multivariable analysis, high interleukin 8 (IL-8) levels were associated with poor survival in both treatment groups, while higher carcinoembryonic antigen (CEA) and hypoxia induced factor 1 alpha (HIF-1alpha) levels were associated with worse survival in patients treated with erlotinib. Complete results are available in S1 and S2 Tables.

**Fig 2:**
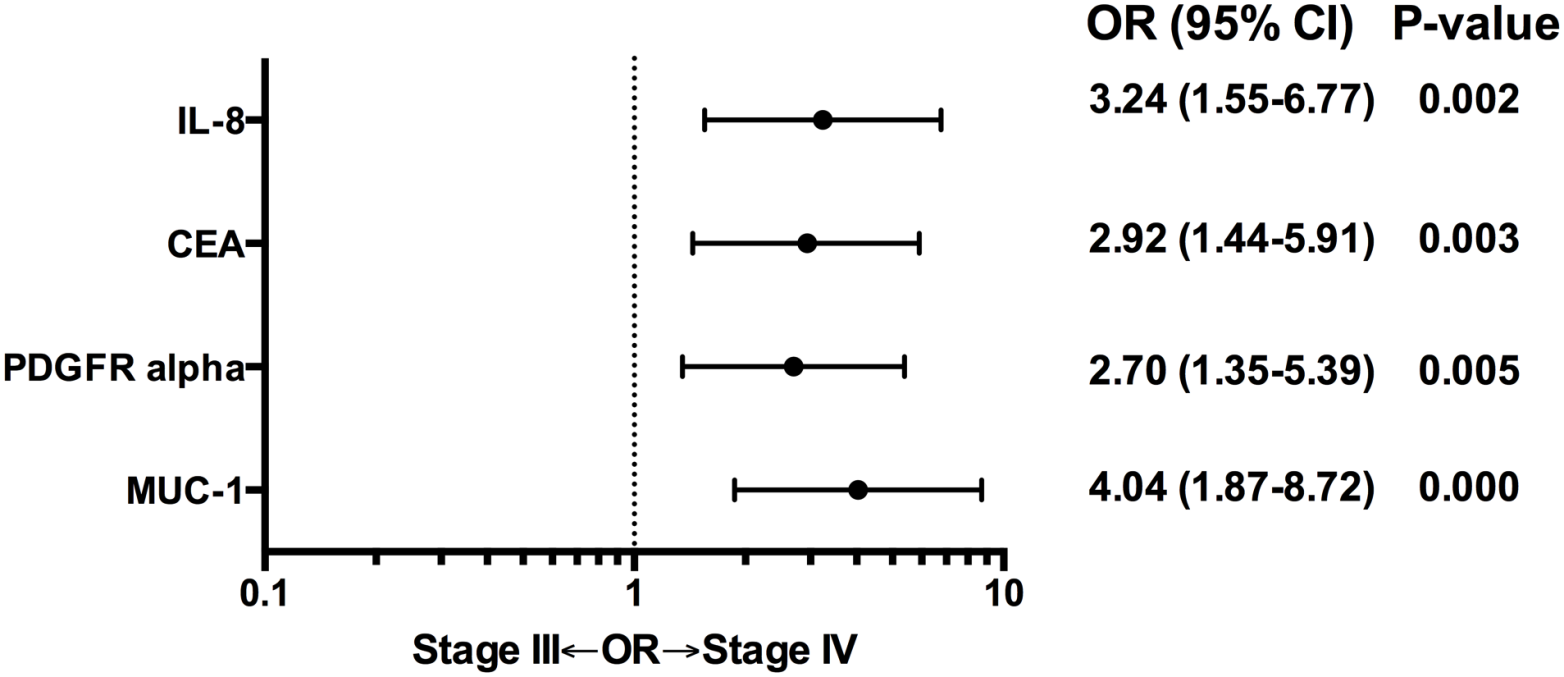
Odds ratios (OR) of having stage III disease (locally advanced) for biomarker concentration, according to median levels (low vs. high). ORs and p-values are shown for biomarkers that were significantly associated (p<0.05). Results shown are from the validation cohort.

Twenty-four percent of patients enrolled on PA.3 had stage III (locally advanced) rather than metastatic disease (stage IV). In a separate analysis, we examined whether or not biomarker concentration (greater than median) was associated with stage (III vs. IV). Significantly more patients with high IL-8, CEA, platelet derived growth factor alpha (PFGFRalpha), and mucin 1 (MUC-1) levels had stage IV metastatic disease.(Fig 2). Results shown are from the validation cohort.

**Fig 3:**
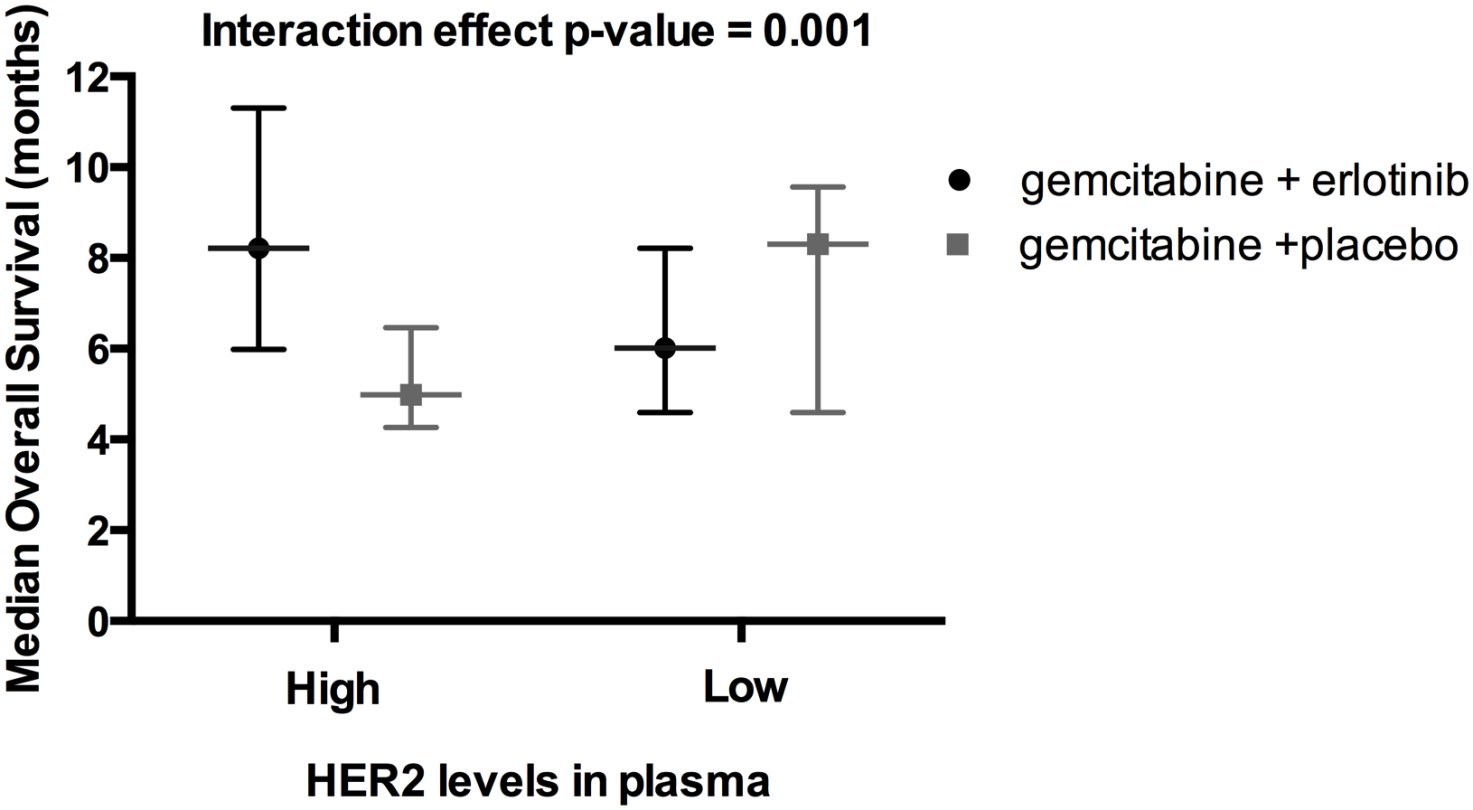
Erlotinib sensitivity is associated with Her2 expression. Median overall survival (with 95% CI) was improved with erlotnib treatment in patients with higher than median levels of plasma HER2 (8.2 vs. 5.0 months) but not in patients with lower plasma HER2 (6.0 vs. 8.3 months).

Finally, we sought to identify biomarkers that predicted for a response to therapy by measuring the interaction effect between erlotinib treatment, biomarker level, and OS. In a multivariable analysis, where biomarker levels were analyzed by being greater or less than the median, none were significantly associated with erlotinib response in both the training and validation sets. However, when the level of each marker was examined as a continuous variable and the statistical constraints were relaxed to p<0.1 for the training set and p<0.05 in the validation set, receptor tyrosine-protein kinase erbB-2 (HER2) alone was identified as having a significant interaction effect with erlotinib response (HR for interaction 0.95, 95% CI 0.72–1.26, p = 0.08 in training cohort and 0.7, 95% CI 0.5–0.99, p = 0.004 in validation cohort). In the validation cohort, patients with HER2 above the median had improved survival when treated with erlotinib (median OS 8.2 vs. 5 months, HR 0.36, 95% CI 0.21–0.63, p<0.0001) whereas no significant difference between erlotinib and placebo was found from patients with HER2 2 levels lower than median (median OS 6.0 vs. 8.3 months, HR 1.28, 95% CI 0.8–2.1, p = 0.3) (Fig 3).

## Discussion

NCIC CTG PA.3 proved its hypothesis that erlotinib plus gemcitabine results in improved OS compared to gemcitabine alone for locally advanced or metastatic PDCA, however median OS in the experimental and control arms differed by only 0.3 months (6.2 versus 5.9). The aims of this current study were to identify biomarkers that were prognostic in either arm, correlative to metastatic versus locally advanced disease, or predictive for improved survival in patients treated with erlotinib versus placebo when added to gemcitabine.

The plasma proteins whose levels were associated with survival in our study, IL-6 [12], IL-8 [13], CEA [14], and HIF-1 alpha [15, 16], have been previously identified as prognostic in locally advanced or metastatic pancreatic cancer. HIF-1 alpha, a transcription factor critical to hypoxia signaling, is not secreted and is presumably detectable in plasma due to tumor lysis. IL-6 [17] and IL-8 [18], which may be secreted from tumor or stromal tissues, are hypoxia induced proteins, suggesting that pancreatic cancer may propagate through hypoxia signaling via HIF-1 alpha, though their role in promoting disease progression is unclear. IL-8 and CEA were also more likely to be elevated in patients with metastatic disease, as were, MUC-1 and PDGFRalpha. Such biomarkers could be used to identify occult metastatic disease in patients who would then be optimally treated with systemic chemotherapy, omitting aggressive local therapy. MUC-1 is overexpressed in pancreatic cancers and has been shown to associate with HIF-1 alpha to drive the expression of hypoxia-induced oncogenes, including PDGF [19, 20], whose receptors, PDGFRalpha and PDGFRbeta regulate PDCA cell migration and metastasis [21, 22].

HER2 was identified as a potentially predictive for erlotinib responsiveness in our study. This is the first clinical example to link disease response to erlotinib with a blood biomarker. Recently, *HER2* was shown to be amplified in 2% of 469 non-pretreated PDCA tumors [23]. HER2 signaling, which is inhibited by erlotinib [24], has been targeted in HER2+ PDCA, assessed by immunohistochemistry, using trastuzumab, in at least 2 previous phase II trials [25, 26], neither of which showed favorable results compared to historical outcomes. It has been recognized that HER2 amplification should be verified using FISH or other methods as opposed to IHC alone [27], which may have led to the previous negative trials. We hypothesize that the modest survival benefit of adding erlotinib to gemcitabine in the PA.3 trial may have been enhanced by using biomarkers such as HER2 to select for patients most likely to respond to erlotinib.

CA 19–9 was not identified as a prognostic biomarker in this study, unlike previous reports [28–31]. One possibility for this result is that in the current study, PLA was used only to report relative quantities of proteins in this highly multi-plexed assay. Analyzing CA 19–9 by a median cutoff value instead of an absolute protein concentration may have affected these results. A prior study using PLA found that although CA 19–9 levels were 16-fold higher in pancreatic patients compared to normal controls, within this population of locally advanced patients, CA 19–9 was also not not prognostic of outcome.[8].

Our study has several limitations. We measured a relatively small number of proteins, and, it is not clear that all PLA probes were able to detect their target with sufficient dynamic range. Because of limited tissue samples, we were not able to confirm whether the markers identified were also over-expressed in tissue and, likewise, because of limited availability of plasma samples, we are unable to verify our results by another method such as enzyme-linked immunosorbent assay (ELISA). However, the accuracy of PLA has been verified via ELISA in prior studies, and the markers that were identified in the current study have been implicated by others as being prognostic for PDCA outcomes. Finally, the recently reported large randomized trial in pancreatic cancer (LAP07 study)l failed to confirm the benefit of erlotinib for PDCA, [32] highlighting the need for predictive biomarkers for erlotinib and other therapies for the treatment of this challenging malignancy.

## Conclusions

We identified several biomarkers that were associated with survival and disease stage in PDCA. Her2 may also be predictive of a response to erlotinib. Further studies are necessary to confirm these results in an independent dataset.

## Supporting Information

S1 Table Results of univariate and multivariable analyses, for gemcitabine plus placebo and gemcitabine plus erlotinib cohorts, including validation and training sets, of the association between biomarker levels and survival. (DOCX)

S2 Table Details of Multivariable Analyses for the biomarkers identified as being significantly correlated (p<0.05) to overall survival (data from validation samples). Results shown indicate the correlation for each variable to survival: HR (CI) and p value.
(DOCX)
