# Supplementary material for: A Novel Biomarker Panel Examining Response to Gemcitabine with or without Erlotinib for Pancreatic Cancer Therapy in NCIC Clinical Trials Group PA.3
Source: PLoS One. 2016 Jan 25;11(1):e0147995. doi: 10.1371/journal.pone.0147995 (PMC4725948; doi:10.1371/journal.pone.0147995)
Supplement: S1 Table — (DOCX) [file pone.0147995.s001.docx]

|  | Univariate | | | | Multivariate | | | |
| --- | --- | --- | --- | --- | --- | --- | --- | --- |
| Biomarker | Training | | Validation | | Training | | Validation | |
|  | HR (CI) | p | HR (CI) | p | HR (CI) | p | HR (CI) | p |
| cxcl-6 | 0.82 (0.59,1.20) | 0.313 | 1.15 (0.78,1.69) | 0.481 | 0.77 (0.51,1.16) | 0.209 | 1.30 (0.85,2.00) | 0.231 |
| CEA | 1.41 (0.95,2.04) | 0.82 | 2.17 (1.47,3.23) | **<0.0001** | 1.25 (0.83,1.89) | 0.283 | 1.69 (1.11,2.56) | **0.014** |
| CA 19-9 | 0.83 (0.57,1.22) | 0.342 | 1.27 (0.86,1.89) | 0.210 | 0.99 (0.65,1.52) | 0.948 | 1.19 (0.79,1.82) | 0.403 |
| HIF-1 alpha | 1.59 (1.09,2.33) | **0.016** | 1.79 (1.20,2.63) | **0.004** | 1.23 (0.83,1.85) | 0.301 | 1.85 (1.22,2.78) | **0.004** |
| SPARC | 0.65 (0.44,0.96) | **0.031** | 1.09 (0.73,1.61) | 0.688 | 0.63 (0.42,0.94) | **0.024** | 1.28 (0.84,1.96) | 0.248 |
| MMP-1 | 1.09 (0.73,1.61) | 0.695 | 1.27 (0.85,1.89) | 0.253 | 1.06 (0.69,1.61) | 0.792 | 1.37 (0.90,2.08) | 0.141 |
| EGFR | 0.68 (0.46,1.01) | 0.056 | 1.08 (0.71,1.61) | 0.738 | 0.72 (0.48,1.10) | 0.125 | 0.95 (0.62,1.47) | 0.834 |
| IL-6 | 1.67 (1.14,2.50) | **0.009** | 1.64 (1.10,2.44) | **0.014** | 1.59 (1.03,2.44) | **0.035** | 1.56 (1.00,2.44) | 0.050 |
| OPN | 0.63 (0.40,0.99) | **0.042** | 1.35 (0.81,2.22) | 0.246 | 0.62 (0.38,0.99) | **0.047** | 1.25 (0.72,2.17) | 0.431 |
| PDK1 | 0.81 (0.54,1.19) | 0.277 | 1.04 (0.70,1.56) | 0.833 | 0.75 (0.50,1.12) | 0.160 | 0.95 (0.62,1.47) | 0.821 |
| IL-8 | 2.04 (1.37,3.03) | **<0.0001** | 1.75 (1.18,2.63) | **0.005** | 1.72 (1.12,2.56) | **0.011** | 1.96 (1.27,3.03) | **0.003** |
| IL-7 | 0.85 (0.57,1.25) | 0.405 | 1.32 (0.88,1.96) | 0.177 | 0.90 (0.60,1.35) | 0.610 | 1.30 (0.85,1.96) | 0.226 |
| Reg IV | 1.22 (0.82,1.79) | 0.332 | 1.20 (0.81,1.79) | 0.371 | 1.11 (0.73,1.67) | 0.635 | 1.14 (0.72,1.79) | 0.576 |
| MSN | 1.06 (0.70,1.61) | 0.761 | 1.96 (1.22,3.13) | **0.004** | 1.01 (0.65,1.56) | 0.967 | 1.75 (0.98,3.13) | 0.059 |
| CXCL-9 | 1.00 (0.67,1.52) | 0.982 | 1.30 (0.86,1.96) | 0.206 | 1.15 (0.75,1.79) | 0.521 | 1.67 (1.05,2.63) | **0.028** |
| IGF-2 | 1.11 (0.74,1.69) | 0.605 | 1.06 (0.74,1.64) | 0.762 | 1.10 (0.70,1.69) | 0.684 | 0.96 (0.60,1.54) | 0.872 |
| cPAI | 1.08 (0.73,1.56) | 0.721 | 0.75 (0.51,1.11) | 0.147 | 1.06 (0.70,1.61) | 0.763 | 0.75 (0.50,1.14) | 0.168 |
| VEGEFD | 0.85 (0.58,1.25) | 0.402 | 1.04 (0.68,1.61) | 0.853 | 0.97 (0.65,1.45) | 0.878 | 1.28 (0.83,2.00) | 0.266 |
| IL-12 | 1.05 (0.71,1.54) | 0.799 | 1.35 (0.87,2.08) | 0.184 | 0.96 (0.65,1.43) | 0.846 | 1.61 (1.01,2.56) | 0.044 |
| ip-10 | 0.78 (0.53,1.15) | 0.212 | 1.43 (0.92,2.22) | 0.112 | 0.81 (0.53,1.25) | 0.338 | 1.32 (0.83,2.08) | 0.240 |
| Her 2 | 1.18 (0.80,1.72) | 0.413 | 1.75 (1.11,2.78) | **0.014** | 1.12 (0.76,1.67) | 0.550 | 1.89 (1.18,3.03) | **0.009** |
| Trappin 2 | 0.69 (0.47,1.01) | 0.057 | 1.25 (0.85,1.85) | 0.248 | 0.60 (0.39,0.92) | **0.018** | 1.27 (0.81,1.96) | 0.297 |
| MMP-2 | 1.23 (0.84,1.82) | 0.282 | 1.52 (1.01,2.22) | **0.041** | 1.35 (0.92,2.00) | 0.127 | 1.41 (0.93,2.13) | 0.099 |
| CXCL-1 | 1.08 (0.72,1.59) | 0.726 | 1.54 (1.04,2.27) | **0.029** | 1.16 (0.76,1.75) | 0.490 | 1.59 (1.04,2.38) | **0.030** |
| Axl | 0.58 (0.39,0.87) | **0.008** | 0.92 (0.62,1.37) | 0.688 | 0.66 (0.44,1.01) | 0.057 | 0.83 (0.54,1.27) | 0.369 |
| PF4 | 0.50 (0.33,0.75) | **0.001** | 0.98 (0.66,1.47) | 0.942 | 0.48 (0.31,0.74) | **0.001** | 0.93 (0.61,1.41) | 0.742 |
| TGFbeta | 0.93 (0.62,1.37) | 0.693 | 1.20 (0.81,1.82) | 0.363 | 0.99 (0.64,1.54) | 0.967 | 1.16 (0.75,1.79) | 0.514 |
| MUC-1 | 1.25 (0.84,1.85) | 0.270 | 2.04 (1.37,3.13) | **<0.0001** | 1.05 (0.68,1.64) | 0.829 | 2.38 (1.52,3.70) | **<0.0001** |
| MMP-3 | 0.78 (0.52,1.18) | 0.232 | 1.43 (0.96,2.17) | 0.075 | 0.64 (0.41,0.98) | **0.040** | 1.20 (0.76,1.89) | 0.437 |
| IGFBP-2 | 0.71 (0.48,1.06) | 0.098 | 0.88 (0.60,1.33) | 0.566 | 0.81 (0.53,1.23) | 0.325 | 0.87 (0.56,1.33) | 0.510 |
| IGF-2 | 0.99 (0.67,1.45) | 0.953 | 1.25 (0.84,1.85) | 0.266 | 1.11 (0.74,1.67) | 0.628 | 1.04 (0.67,1.61) | 0.872 |
| BMP 2-4 | 1.10 (0.75,1.61) | 0.625 | 1.03 (0.69,1.52) | 0.891 | 1.11 (0.74,1.67) | 0.623 | 1.09 (0.71,1.67) | 0.687 |
| PDGFRa | 1.04 (0.70,1.52) | 0.851 | 1.19 (0.81,1.75) | 0.365 | 0.88 (0.58,1.35) | 0.574 | 1.39 (0.89,2.17) | 0.147 |
| MMP-7 | 1.10 (0.75,1.61) | 0.624 | 1.89 (1.27,2.86) | **0.002** | 0.98 (0.65,1.47) | 0.911 | 1.54 (0.97,2.38) | 0.066 |
| IGFBP-3 | 0.67 (0.45,0.98) | **0.040** | 1.33 (0.91,2.00) | 0.142 | 0.77 (0.51,1.16) | 0.212 | 1.27 (0.83,1.92) | 0.263 |
| Gas6 | 1.18 (0.80,1.72) | 0.411 | 1.18 (0.80,1.72) | 0.402 | 1.14 (0.75,1.72) | 0.537 | 1.32 (0.86,2.00) | 0.205 |

Table 1 supplemental: gemcitabine and placebo arm

|  | Univariate | | | | Multivariate | | | |
| --- | --- | --- | --- | --- | --- | --- | --- | --- |
| Biomarker | Training | | Validation | | Training | | Validation | |
|  | HR (CI) | p | HR (CI) | p | HR (CI) | p | HR (CI) | p |
| cxcl-6 | 0.76 (0.52,1.11) | 0.154 | 1.22 (0.78,1.89) | 0.387 | 0.75 (0.50, 1.12) | 0.157 | 1.52 (0.91,2.50) | 0.109 |
| CEA | 1.69 (1.16,2.50) | **0.006** | 1.52 (0.96,2.38) | 0.073 | 1.69 (1.11,2.56) | **0.013** | 1.67 (1.02,2.70) | **0.042** |
| CA 19-9 | 1.11 (0.76,1.61) | 0.584 | 0.69 (0.45,1.06) | 0.091 | 1.25 (0.83,1.89) | 0.275 | 0.72 (0.46,1.15) | 0.170 |
| HIF-1 alpha | 1.56 (1.08,2.27) | **0.019** | 1.54 (0.98,2.38) | 0.057 | 1.59 (1.06,2.33) | **0.022** | 1.69 (1.05,2.70) | **0.031** |
| SPARC | 0.91 (0.62,1.32) | 0.612 | 0.97 (0.62,1.52) | 0.884 | 0.69 (0.46,1.03) | 0.070 | 0.93 (0.57,1.49) | 0.752 |
| MMP-1 | 1.52 (1.02,2.22) | **0.036** | 0.98 (0.63,1.56) | 0.949 | 1.64 (1.09,2.50) | **0.019** | 1.10 (0.67,1.82) | 0.701 |
| EGFR | 1.32 (0.90,1.92) | 0.158 | 0.92 (0.58,1.43) | 0.696 | 1.15 (0.08,1.72) | 0.492 | 0.79 (0.48,1.30) | 0.355 |
| IL-6 | 1.75 (1.19,2.56) | **0.004** | 1.27 (0.80,2.00) | 0.315 | 1.79 (1.18,2.70) | **0.006** | 1.43 (0.88,2.33) | 0.147 |
| OPN | 1.00 (0.65,1.54) | 0.996 | 0.91 (0.53,1.54) | 0.726 | 1.04 (0.66,1.64) | 0.876 | 1.39 (0.75,2.56) | 0.294 |
| PDK1 | 1.22 (0.84,1.79) | 0.292 | 0.68 (0.42,1.10) | 0.111 | 1.22 (0.82,1.82) | 0.333 | 1.12 (0.57,2.17) | 0.737 |
| IL-8 | 1.82 (1.23,2.63) | **0.002** | 1.89 (1.19,3.03) | **0.006** | 1.79 (1.19,2.70) | **0.006** | 2.44 (1.45,4.00) | **0.001** |
| IL-7 | 0.70 (0.47,1.02) | 0.064 | 0.98 (0.62,1.54) | 0.921 | 0.67 (0.45,0.99) | **0.045** | 1.10 (0.67,1.82) | 0.699 |
| Reg IV | 1.75 (1.19,2.63) | 0.004 | 0.94 (0.60,1.49) | 0.796 | 1.82 (1.19,2.78) | **0.005** | 0.88 (0.51,1.52) | 0.650 |
| MSN | 1.00 (0.65,1.52) | 0.989 | 0.85 (0.51,1.41) | 0.517 | 0.92 (0.58,1.43) | 0.699 | 1.05 (0.57,1.92) | 0.881 |
| CXCL-9 | 1.15 (0.78,1.72) | 0.482 | 1.22 (0.76,1.96) | 0.411 | 1.12 (0.74,1.72) | 0.578 | 1.33 (0.78,2.33) | 0.286 |
| IGF-2 | 0.81 (0.55,1.20) | 0.295 | 0.51 (0.32,0.81) | **0.004** | 0.80 (0.05,1.22) | 0.302 | 0.46 (0.27,0.76) | **0.003** |
| cPAI | 0.91 (0.63,1.32) | 0.608 | 0.83 (0.54,1.30) | 0.424 | 0.82 (0.56,1.20) | 0.319 | 0.83 (0.53,1.33) | 0.449 |
| VEGEFD | 0.94 (0.64,1.39) | 0.766 | 0.61 (0.37,1.01) | 0.050 | 0.94 (0.64,1.41) | 0.788 | 0.65 (0.39,1.09) | 0.101 |
| IL-12 | 0.66 (0.45,0.97) | 0.033 | 0.90 (0.56,1.45) | 0.676 | 0.65 (0.44,0.96) | **0.033** | 1.04 (0.61,1.79) | 0.883 |
| ip-10 | 1.30 (0.88,1.92) | 0.186 | 0.83 (0.51,1.35) | 0.468 | 1.25 (0.08,1.89) | 0.278 | 0.86 (0.51,1.47) | 0.584 |
| Her 2 | 0.98 (0.67,1.45) | 0.930 | 0.69 (0.43,1.12) | 0.133 | 0.86 (0.57,1.30) | 0.478 | 0.68 (0.39,1.19) | 0.182 |
| Trappin 2 | 0.98 (0.67,1.43) | 0.913 | 1.02 (0.66,1.59) | 0.942 | 0.94 (0.63,1.41) | 0.766 | 0.85 (0.52,1.39) | 0.518 |
| MMP-2 | 1.03 (0.70,1.52) | 0.890 | 0.81 (0.52,1.27) | 0.348 | 1.01 (0.68,1.52) | 0.961 | 0.73 (0.44,1.20) | 0.216 |
| CXCL-1 | 0.94 (0.65,1.39) | 0.762 | 0.92 (0.59,1.43) | 0.697 | 0.88 (0.58,1.32) | 0.535 | 1.12 (0.70,1.79) | 0.643 |
| Axl | 0.96 (0.66,1.39) | 0.844 | 0.58 (0.37,0.91) | 0.016 | 0.96 (0.65,1.41) | 0.828 | 0.65 (0.39,1.06) | 0.088 |
| PF4 | 0.59 (0.40,0.87) | **0.007** | 1.10 (0.70,1.69) | 0.682 | 0.65 (0.04,0.96) | **0.031** | 1.22 (0.76,1.92) | 0.407 |
| TGFbeta | 0.98 (0.68,1.43) | 0.921 | 0.87 (0.56,1.37) | 0.548 | 0.96 (0.66,1.41) | 0.858 | 0.84 (0.50,1.41) | 0.513 |
| MUC-1 | 1.43 (0.97,2.08) | 0.066 | 1.54 (0.99,2.38) | 0.055 | 1.27 (0.85,1.85) | 0.250 | 1.69 (1.01,2.86) | **0.047** |
| MMP-3 | 0.71 (0.49,1.04) | 0.081 | 0.84 (0.53,1.32) | 0.449 | 0.64 (0.43,0.95) | **0.027** | 0.85 (0.49,1.45) | 0.547 |
| IGFBP-2 | 0.74 (0.51,1.08) | 0.112 | 0.58 (0.37,0.92) | 0.019 | 0.65 (0.44,0.97) | **0.035** | 0.66 (0.39,1.11) | 0.119 |
| IGF-2 | 1.23 (0.84,1.79) | 0.287 | 0.79 (0.50,1.25) | 0.308 | 1.20 (0.80,1.82) | 0.365 | 0.81 (0.49,1.33) | 0.408 |
| BMP 2-4 | 0.99 (0.68,1.45) | 0.973 | 0.88 (0.56,1.39) | 0.596 | 1.03 (0.69,1.54) | 0.863 | 0.94 (0.56,1.59) | 0.829 |
| PDGFRa | 1.64 (1.11,2.44) | 0.011 | 1.49 (0.94,2.38) | 0.088 | 1.47 (0.97,2.22) | 0.071 | 1.33 (0.77,2.27) | 0.310 |
| MMP-7 | 1.67 (1.14,2.44) | **0.009** | 1.28 (0.82,2.00) | 0.270 | 1.79 (1.18,2.78) | **0.007** | 1.59 (0.92,2.70) | 0.098 |
| IGFBP-3 | 1.14 (0.78,1.64) | 0.516 | 1.03 (0.65,1.61) | 0.909 | 1.01 (0.66,1.54) | 0.973 | 1.09 (0.65,1.82) | 0.736 |
| Gas6 | 0.96 (0.66,1.39) | 0.821 | 1.14 (0.74,1.75) | 0.549 | 1.00 (0.68,1.47) | 0.983 | 1.28 (0.78,2.08) | 0.330 |

Table 1 supplemental: gemcitabine and erlotinib arm
