# Supplementary material for: A Novel Biomarker Panel Examining Response to Gemcitabine with or without Erlotinib for Pancreatic Cancer Therapy in NCIC Clinical Trials Group PA.3
Source: PLoS One. 2016 Jan 25;11(1):e0147995. doi: 10.1371/journal.pone.0147995 (PMC4725948; doi:10.1371/journal.pone.0147995)
Supplement: S2 Table — Results shown indicate the correlation for each variable to survival: HR (CI) and p value. (DOCX) [file pone.0147995.s002.docx]

**S2 Table: Details of Multivariable Analyses for the biomarkers identified as being significantly correlated (p<0.05) to overall survival (data from validation samples).** Results shown indicate the correlation for each variable to survival: HR (CI) and p value.

|  | **Biomarker**  **(Low vs. High)** | **Age**  **(<70 vs. ≥ 70)** | **Sex**  **(Female vs. Male)** | **Race**  **(White vs. non-white)** | **ECOG Performance status (0-1 vs. 2)** | **Pain intensity**  **(<=20 vs. >20)** | **Stage**  **(III vs. IV)** |
| --- | --- | --- | --- | --- | --- | --- | --- |
| **(1) Gemecitabine+Placebo** | | | | | | | |
| **IL-8** | 1.96 (1.27-3.03) p=0.003 | 1.06 (0.67-1.69) p=0.79 | 1.47 (0.97-2.22) p=0.07 | 0.96 (0.53-1.72) p=0.900 | 4.00 (2.27-7.14) p=<0.0001 | 0.92 (0.60-1.41) p=0.70 | 2.78 (1.59-4.76) p=0.0003 |
| **(2) Gemecitabine+Erlotinib** | | | | | | | |
| **HIF-1 alpha** | 1.69 (1.05-2.70) p=0.031 | 1.47 (0.91-2.38) p=0.12 | 1.09 (0.68-1.72) p=0.72 | 1.08 (0.58-2.00) p=0.81 | 1.15 (0.63-2.13) p=0.65 | 1.85 (1.11-3.13) p=0.02 | 1.27 (0.65-2.44) p=0.49 |
| **IL-8** | 1.96 (1.16-3.33) p=0.012 | 1.43 (0.86-2.33) p=0.17 | 1.10 (0.68-1.75) p=0.70 | 1.12 (0.60-2.13) p=0.71 | 0.92 (0.47-1.79) p=0.79 | 2.00 (1.16-3.33) p=0.01 | 1.04 (0.49-2.22) p=0.92 |
| **CEA** | 1.67 (1.02-2.70) p=0.042 | 1.45 (0.90-2.33) p=0.13 | 1.09 (0.68-1.72) p=0.71 | 1.05 (0.57-1.96) p=0.86 | 1.18 (0.63-2.17) p=0.61 | 1.85 (1.11-3.13) p=0.02 | 1.19 (0.61-2.33) p=0.60 |
| **(3) All** | | | | | | | |
| **HIF-1 alpha** | 1.72 (1.27-2.33) p=0.001 | 1.18 (0.85-1.61) p=0.33 | 1.25 (0.93-1.69) p=0.14 | 0.95 (0.63-1.43) p=0.82 | 1.79 (1.20-2.63) p=0.004 | 1.27 (0.93-1.72) p=0.14 | 2.00 (1.33-2.94) p=0.001 |
| **IL-8** | 1.61 (1.18-2.22) p=0.003 | 1.16 (0.83-1.64) p=0.37 | 1.25 (0.93-1.72) p=0.15 | 0.97 (0.64-1.47) p=0.89 | 1.67 (1.11-2.50) p=0.01 | 1.25 (0.91-1.69) p=0.17 | 1.89 (1.23-2.94) p=0.003 |
| **CEA** | 1.72 (1.27-2.33) p=0.001 | 1.16 (0.84-1.59) p=0.36 | 1.22 (0.90-1.64) p=0.19 | 0.92 (0.61-1.39) p=0.67 | 1.72 (1.16-2.50) p=0.006 | 1.25 (0.93-1.69) p=0.14 | 1.85 (1.23-2.78) p=0.003 |
| **IL-6** | 1.41 (1.03-1.92) p=0.03 | 1.06 (0.76-1.49) p=0.71 | 1.27 (0.93-1.72) p=0.14 | 1.00 (0.66-1.52) p=0.99 | 1.75 (1.18-2.63) p=0.006 | 1.32 (0.95-1.82) p=0.09 | 2.00 (1.32-3.03) p=0.001 |
